# Successful combination treatment with azacitidine and venetoclax as a bridging therapy for third allogenic stem cell transplantation in a patient with 11q23/MLL‐rearranged complex karyotype acute myeloid leukemia

**DOI:** 10.1002/jha2.630

**Published:** 2022-12-26

**Authors:** Taro Edahiro, Hiroshi Ureshino, Ren Chishaki, Keita Fujino, Tatsuji Mino, Tetsumi Yoshida, Noriyasu Fukushima, Tatsuo Ichinohe

**Affiliations:** ^1^ Department of Hematology and Oncology Research Institute for Radiation Biology and Medicine Hiroshima University Hiroshima Japan; ^2^ Next Generation Development of Genome and Cellular Therapy Program Research Institute for Radiation Biology and Medicine (RIRBM) Hiroshima University Hiroshima Japan; ^3^ Department of Internal Medicine Karatsu Red Cross Hospital Karatsu Japan

**Keywords:** 11q23/MLL‐rearranged acute myeloid leukemia, azacitidine, BCL‐2, venetoclax

## Abstract

Translocation t(6;11) occurs in approximately 5% of patients with acute myeloid leukemia (AML) corresponding to 11q23/mixed lineage leukemia (MLL) rearrangement. The AF6 gene on chromosome 6q27 is the fusion partner of the MLL gene on 11q23 in t(6;11), which results in a poor prognosis. The case of a patient with 11q23/MLL‐rearranged AML who successfully underwent a third allogeneic stem cell transplantation after treatment with azacitidine (AZA) and venetoclax (VEN) is presented in this article. This report suggests that a combination of AZA and VEN is an effective therapeutic approach for relapsed and refractory MLL‐rearranged AML.

## 1 INTRODUCTION

Allogeneic hematopoietic stem cell transplantation (allo‐SCT) is an effective treatment for patients with acute myeloid leukemia (AML); however, posttransplant relapse occurs in a considerable proportion of patients with a generally dismal prognosis [1]. Approximately 5% of patients with AML present with translocation *t*(6;11), which corresponds to the 11q23/mixed lineage leukemia (MLL) rearrangement. The MLL gene on 11q23 fuses with the AF6 gene on chromosome 6q27, which results in a poor prognosis [2] can be defined as an adverse prognostic cytogenetic abnormality [3]. In this study, we report the case of a patient with 11q23/MLL‐rearranged complex karyotype AML who successfully underwent a third allo‐SCT for second relapse.

## 2 CASE PRESENTATION

A 22‐year‐old man who was previously diagnosed with acute monoblastic leukemia at 17 years of age was admitted to the hospital. The patient had undergone allo‐SCT twice; unrelated bone marrow (BM) transplantation during the first complete remission (CR); and cord blood transplantation (CBT) for leukemia relapse. Unfortunately, a second leukemia relapse occurred 11 months after CBT. BM aspiration revealed normocellularity with 7% leukemic blasts. G‐banded metaphase BM chromosome analysis at initial diagnosis showed 47, XY, −2, +add(8)(p11.2), del(11)(q?), mar + 1[9], 48, idem, +add(8)[9], 49, idem, +add(8) × 2 [2], and that of BM cells at relapse showed 46XY, inv(2)(p11.2q13), t(6;11)(q27;q23.3)[2], and 46XX [18], respectively. The results of the fluorescence in situ hybridization (FISH) for the detection of MLL gene rearrangement indicated the existence of 7% of MLL gene‐rearranged cells. This suggests that additional MLL gene rearrangement was involved in the leukemia relapse. Gemtuzumab ozogamicin (GO) monotherapy (3 mg/m^2^) was subsequently initiated as salvage chemotherapy. After six cycles of GO, hematological CR and complete donor chimerism were achieved. Furthermore, the patient was placed under observation.

BM aspirate indicated normocellularity with 11.6% myeloblasts in the follow‐up after 16 months. The G‐banded metaphase analysis showed a more complex karyotype; 47, XY, add (1)(p11), del(1)(p?), −2, del(2)(q?), t(3;15)(p21;q26), −5, −6, −7, der(11)?t(6;11)(q27;q23.3), −13, +der(?)t(?;2)(?;q21), +mar1, +mar2, +mar3, +mar4, +mar5[5]46, and XX [15]. FISH results revealed the existence of 37% of MLL rearrangements, whereas the reverse transcription–polymerase chain reaction (RT‐PCR) revealed no mutation of FLT3 internal tandem duplications. GO treatment was resumed, and then hematological CR was achieved after the first course of the GO treatment. On the other hand, FISH results indicated 7% MLL rearrangement. Subsequently, a combination treatment of AZA (75 mg/m^2^) and VEN (400 mg/day) was initiated as salvage chemotherapy. As AZA and VEN were well tolerated, no treatment interruption or delay were observed (grade 3 neutropenia was developed only once, and no other severe adverse events were observed). After three cycles of AZA and VEN, BM aspiration indicated hematological CR with no MLL rearrangement as determined using FISH. Moreover, no *MLL::AF6* transcript was detected via RT‐PCR in the peripheral blood sample (BM sample could not be assessed due to low DNA quantity). These results demonstrated the three cycles of AZA and VEN most probably yielded molecular CR as suggested by blood cell evaluation by FISH.

**FIGURE 1:**
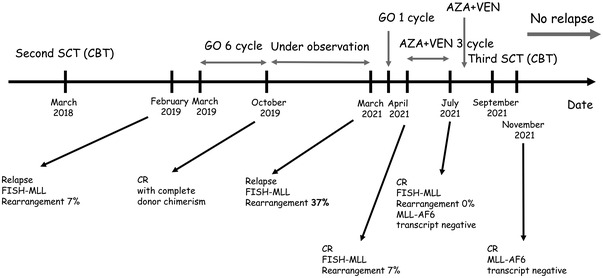
Clinical course AZA, azacitidine; CBT, cord blood transplantation; CR, complete remission; FISH, fluorescence in situ hybridization; GO, gemtuzumab ozogamicin; MLL, mixed lineage leukemia; SCT, hematopoietic stem cell transplantation; VEN, venetoclax

After one additional cycle of the treatment, the patient underwent a third allo‐SCT. The patient received a reduced intensity conditioning regimen of fludarabine (30 mg/m^2^; from day −6 to −3), melphalan (140 mg/m^2^; day −2), and 2Gy (day −1) of total body irradiation followed by the infusion of cord blood cells containing 0.89 × 10^5^ /kg CD34‐positive cells. Graft‐versus‐host disease (GVHD) prophylaxis included tacrolimus (from day −1) and mycophenolate mofetil (from day +1). An interim BM aspiration at day +15 showed the early achievement of complete donor chimerism without residual blasts, and no MLL rearrangement was detected by FISH. Neutrophil engraftment was achieved at day +32. Only grade 1 acute GVHD (skin stage 1, liver 0, and gut 0) which was well manageable was developed. BM aspiration at day +85 showed the CR continuation with complete donor chimerism and no *MLL::AF6* transcript by RT‐PCR. The patient has maintained CR 13 months after transplantation (Figure 1).

## 3 DISCUSSION

The combinations of hypomethylating agents (AZA or decitabine) or low‐dose cytarabine and VEN have been considered the standard treatment strategy for patients with previously untreated or relapsed and refractory AML who are ineligible for allo‐SCT because of its high efficacy and low toxicity rate, especially in patients with adverse cytogenetic abnormality [4, 5]. *MLL::AF4* acute lymphoblastic leukemia (ALL) showed a highly expressed BCL‐2 that is directly regulated by *MLL::AF4* [6]. Moreover, MLL‐rearranged ALL was highly sensitive for VEN treatment [7, 8]. MLL‐rearranged AML was also strongly expressed BCL‐2 similar with MLL‐ALL patients based on publicly available transcriptome data (**GSE13159**, Figure S1). Considering the fact that patients with chronic lymphocytic leukemia (CLL) that is sensitive to VEN are dependent on high BCL‐2 expression in CLL cells, VEN may be sensitive to MLL‐rearranged AML. Hence, the combination treatment of AZA and VEN for MLL‐ rearranged AML is a reasonable treatment strategy. In the present case, AZA and VEN successfully induced molecular CR (subsequently proceeding to allo‐SCT) in a patient who previously received intensive treatment with MLL‐rearranged AML. These findings suggest that AZA and VEN may have an effective therapeutic potential for relapsed and refractory MLL‐rearranged AML, especially in patients who have undergone intensive treatment.

## AUTHOR CONTRIBUTIONS

All authors contributed to patient care. Taro Edahiro, Hiroshi Ureshino, and Tatsuo Ichinohe wrote the manuscript. All authors approved the final version of the manuscript.

## CONFLICTS OF INTEREST

TI received consultant fee from AbbVie, Nippon Shinyaku, Repertoire Genesis, and Nihon Kayaku. TI received grants from AbbVie, Nippon Shinyaku, Repertoire Genesis, Asahi Kasei, Chugai pharmaceutical, CBL Behring, Daiichi Sankyo, Eisai, Kyowa Hakko Kirin, Ono Pharmaceutical, Otsuka Pharmaceutical Sumitomo Dainippon Pharma, and Zenyaku Kogyo. TI received honoraria from Bristol‐Myers Squibb, Eisai, FUJIFILM Wako chemicals, Kyowa Hakko Kirin, Novartis Pharma K.K, Ono pharmaceutical, and Pfizer. No other authors declare potential conflicts of interest (COI).

## ETHICS STATEMENT

All procedures performed in studies involving human participants were in accordance with the ethical standards of the institutional and/or national research committee and with the 1964 Helsinki declaration and its later amendments or comparable ethical standards.

## PATIENT CONSENT STATEMENT

The patient provided informed consent to publish this case report.

## Supporting information

Supporting information

## Data Availability

All data can be accessed by contacting the corresponding author(Ureshino H).
