# Supplementary figures and images for: Successful combination treatment with azacitidine and venetoclax as a bridging therapy for third allogenic stem cell transplantation in a patient with 11q23/MLL‐rearranged complex karyotype acute myeloid leukemia
Source: EJHaem. 2022 Dec 26;4(1):273–5. doi: 10.1002/jha2.630 (PMC9928651; doi:10.1002/jha2.630)

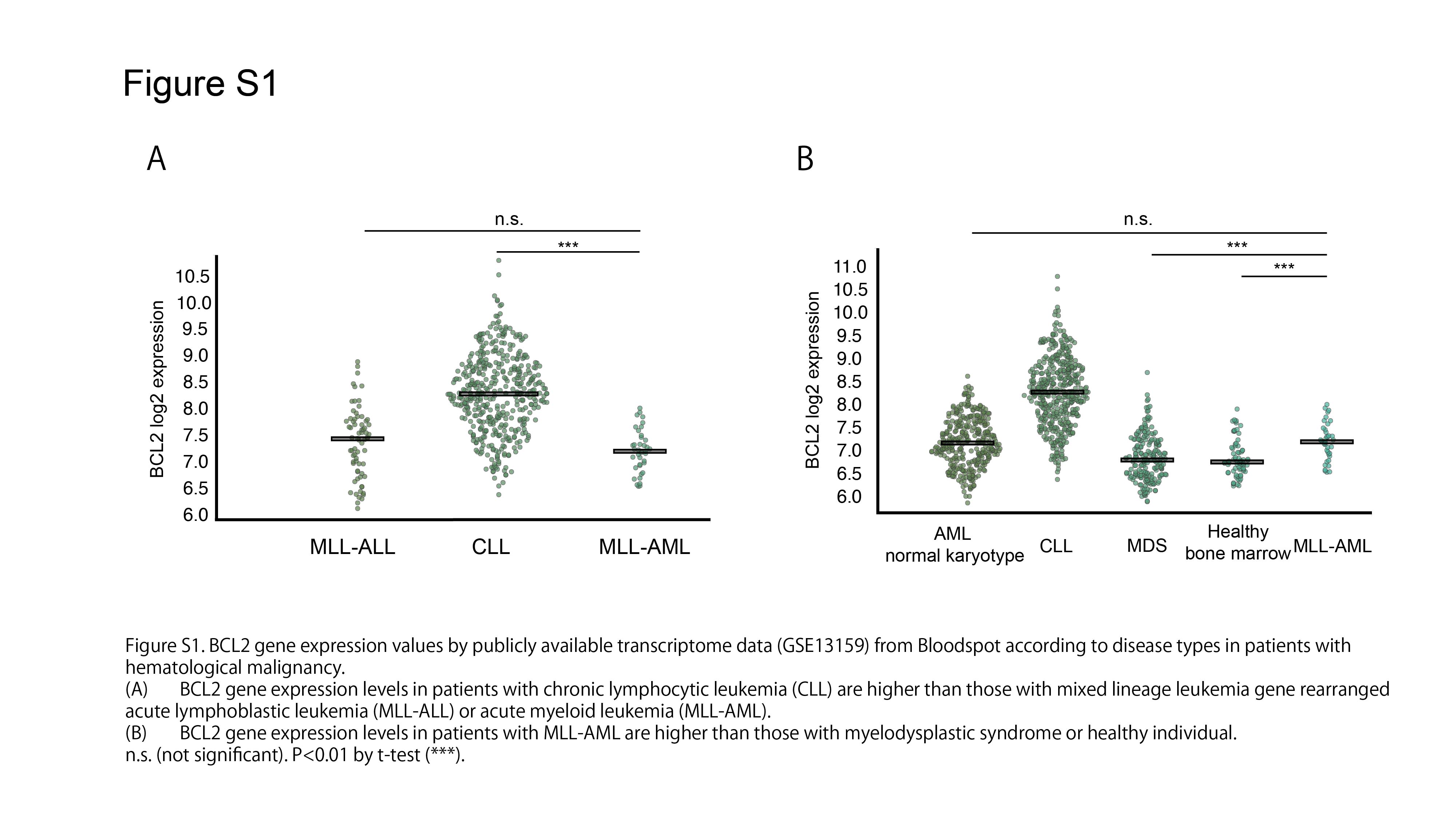

Supplement: Supplementary file 1 — Supporting information [file JHA2-4-273-s001.jpg]
